# Capturing Beneficial Changes to Racehorse Veterinary Care Implemented during the COVID-19 Pandemic

**DOI:** 10.3390/ani11051251

**Published:** 2021-04-26

**Authors:** Deborah Butler, Lois Upton, Siobhan Mullan

**Affiliations:** 1 School of Veterinary Sciences, University of Bristol, Langford BS40 5DU, UK; siobhan.mullan@ucd.ie; 2 British Horseracing Authority, 75 High Holborn, London WC1V 6LS, UK; lupton@britishhorseracing.com

**Keywords:** COVID-19, racehorse veterinary care, telemedicine, connectivity, thematic analysis, telephone interviewing, lockdown

## Abstract

**Simple Summary:**

On 23 March 2020, the UK Government introduced its first nationwide lockdown as part of efforts to reduce the impact of COVID-19. There have since been two more. “Lockdown” control measures meant both racehorse trainers and veterinary surgeons (vets) had to make changes to the way they worked. Beneficial practices which aided veterinary care have been reported anecdotally, as has an increase in the use of electronic communication and information technologies. The aim of this study was to investigate if these claims could be supported by exploring any changes to racehorse veterinary care that occurred due to the implementation of the first COVID-19 lockdown restrictions. Data collection involved carrying out ten semistructured interviews with racehorse trainers and ten equine vets from November 2020 to January 2021. Reflexive thematic analysis was used to try and make sense of how vets and trainers interacted with each other before and during a period of rapid change and how both groups found alternative ways to ensure beneficial veterinary care was not compromised. Data Analysis revealed four themes threading through the data. These were, firstly, the trainer–vet relationship is built upon a good working relationship, secondly, there had been little or no change in the vet–trainer relationship during the first “lockdown” period. Thirdly, when COVID-19 restrictions were in force, more remote consultations took place using images or videos as well as telephone consults, and the fourth and final theme identified the way connectivity and poor-quality images and videos limited their effectiveness.

**Abstract:**

In March 2020, the World Health Organisation called for countries to take urgent and aggressive action against a global pandemic caused by COVID-19. Restrictions were introduced in many countries to reduce transmission of COVID-19 and ultimately deaths. Such restrictions have been colloquially referred to as “lockdown”. Anecdotal evidence of the beneficial practices that facilitated safe veterinary treatment and equine care had been reported together with an increase in the use of electronic communication and information technologies during the first “lockdown”. Thus, the aim of this qualitative study was to capture any beneficial changes to racehorse veterinary care that were implemented during the first “lockdown” period in the UK that lasted from 23 March to 12 May 2020. Ten equine veterinary surgeons who primarily treat racehorses and 10 racehorse trainers were interviewed either by telephone or by videoconferencing. After using thematic analysis from a critical realist social constructionist perspective, four themes were identified. These were, firstly, according to our participants, the trainer–vet relationship is predicated upon a good working relationship, secondly, there had been little or no change in the vet–trainer relationship during the first “lockdown” period. Thirdly, when COVID-19 restrictions were in force, more remote consultations took place using images or videos as well as telephone consults, viewed favourably by both trainers and vets, and finally, intermittent connectivity and poor-quality images and videos limited their effectiveness. In order to fully benefit from the positive changes employed by some vets and trainers in their working relationships, we recommend that rural connectivity is prioritised.

## 1. Introduction

A novel coronavirus, SARS-CoV-2, linked to cases of human viral pneumonia was first reported in Wuhan, China in December 2019, and the disease it causes was subsequently named COVID-19 in March 2020. On 11 March 2020, the World Health Organization (WHO) declared the coronavirus disease (COVID-19) outbreak a global pandemic and an ongoing public health emergency [1]. Restrictions were imposed by many countries, including the UK, where the first case of SARS-CoV-2 transmission inside the United Kingdom (UK) was confirmed on 29 January 2020. The WHO called for countries to take urgent and aggressive action to prevent infections with the aim of reducing transmission of SARS-CoV-2 and reducing the basic reproduction number of the virus (R0). Such restrictions have been colloquially referred to as “lockdown” restrictions [2]. Between 23 March and 21 June, there will have been three “lockdown” periods in the UK [3].

The first “lockdown” restrictions were introduced with immediate effect in the UK (in place 23 March–12 May 2020), focusing on the message of “stay at home.” Measures included quarantining, social distancing and the closure of all nonessential retail business, restaurants and bars [4]. Consequently, “stay at home” orders and physical distancing recommendations have served as potential inhibitors to outdoor recreational activities central to the wellbeing and lifestyles of outdoor enthusiasts, for instance, hiking, biking, running [5] and equine activities such as horse riding [6] and horseracing [7].

British horseracing, the country’s second largest sport behind football in terms of attendances, employment and revenue was not immune to government-enforced “lockdown” restrictions, with racing suspended from 18 March 2020 to 1 June 2020. The “lockdown” brought the National Hunt season (jump racing) to an abrupt halt with it not resuming until 1 July 2020. The start of the traditional flat racing season started on 1 June 2020 instead of at the end of March but was behind “closed doors”, with no public spectators permitted [7]. As in many organisations and businesses, the pandemic has had a wide-ranging impact among all stakeholders in racing, including racecourses, whose tight profit margins have, in some cases, been wiped out and whose sponsors have not had the opportunity to provide corporate hospitality at race meetings. Trainers found themselves reducing the number of horses in full work who were ready to race, so affecting their turnover, caused by a reduction in training fees they received from owners [8]. For some trainers and veterinary practices, being able to furlough staff, that is, make use of the UK Coronavirus Job Retention Scheme, has helped them weather the financial hit their cash flow and turnover has had to endure [9,10].

Introduced on 20 March 2020, the UK Coronavirus Job Retention Scheme formed part of a series of wide-ranging measures brought in to assist businesses and employees through the COVID-19 crisis. In brief, employers were able to contact Her Majesty’s Revenue and Customs (HMRC) to “furlough” their staff. Furloughing involved being in receipt of a grant to cover 80% of wages (up to a total of £2500 per month) for employees who were not working. Rather than being dismissed by being “furloughed”, they were kept on payroll [11]. Racehorse trainers, for instance, were able to furlough staff, introducing different working practices within yards to mitigate the effect of a smaller workforce [12,13]. 

One of the consequences of the pandemic has been the rapid growth in academic interest into the impact of COVID-19. Initially, urgent public health research into the effect of COVID-19 was given prioritised support [14], now, more recently, with the successful creation of a COVID-19 vaccine, research has started to concentrate on the long-term consequences of the pandemic [14,15]. During 2020, there has been a plethora of studies surrounding COVID-19 and the pandemic, which have included for instance, how engineered llama antibodies can neutralize the COVID-19 virus [16], the effect of limiting care home occupancy rates [17], the impact of COVID-19 on bettors [18], human–animal relationships and the effect of COVID-19 during lockdown [19], human–dog relationships during the pandemic [20], companion animals as potential carriers of COVID-19 [21,22] and the effects of COVID-19 on livestock production, where the authors investigated the impact COVID-19 is having on “One welfare” that is “the interconnections between animal welfare, human wellbeing and the environment” [23]. However, at present there would appear to be little academic research on the effect of the pandemic on equine welfare and veterinary care, especially within the horseracing industry. Some veterinary equine practices have published on-line guides for horse owners on how to care for their horse during coronavirus [24], advising clients on, for instance, exercise, feeding and daily care, but there is little that investigates the disruption COVID-19 has had on veterinary advice, diagnosis and treatment of racehorses during the pandemic.

From the beginning of April, the Royal College of Veterinary Surgeons (RCVS) ran short surveys to assess the immediate impact of Covid-19 on veterinary clinical services, specifically from a business and economic perspective with a view to informing further policy decisions [25]. In April 2020, of the 532 practices who completed the first survey, 66% had seen their weekly practice turnover reduce since “lockdown” measures were put in place, with the turnover of the majority of equine practices reduced by over 75% when compared with small animal or mixed practices [26]. However, as “lockdown” restrictions were lifted, by September 2020, practice turnover had either stayed the same or increased for all practice types. In April, 62% of respondents had also furloughed or intended to furlough veterinary surgeons, although furloughing staff had created significant stress on veterinary personnel who continued to work as well as difficulties in creating an acceptable working rota. As rules have changed across the UK, veterinary practices have been adapting their working practices and range of services in line with local, regional and national lockdown measures [27]. As restrictions on working practices have been eased, there has been a large drop in the number of veterinary surgeons furloughed. In September 2020, 10% of practices had veterinary surgeons furloughed compared to 47% in June 2020 [28].

Throughout the COVID-19 crisis, veterinary professionals have been able to work, in line with the UK government’s advice to business. Initially (in March 2020), this work was limited to urgent and emergency services, and services to maintain the food supply chain [29]. Practices were then able to provide services deemed essential for animal health and welfare or public health, including measures to relieve pain and suffering. During the crisis, some veterinary practices had to defer some non-urgent procedures to preserve medical and pharmaceutical supplies. Some countries (e.g., UK, US and FR) have exceptionally allowed remote consultation and prescribing to strike the right balance between providing essential veterinary care for animals and safeguarding the health of the profession and the public [30]. Beneficial practices that facilitated safe veterinary treatment and equine care have been developed and reported anecdotally. For example, in order to promote social distancing of staff, one veterinary surgeon started implanting microchips in foals (required by law for identification purposes) that have an additional temperature sensor, enabling a single carer to take the temperature of a foal, rather than the two people usually required [31]. 

Telemedicine, that is, the use of different types of electronic communication and information technologies, in veterinary practice has been much discussed prior to COVID-19 with only a minority of veterinary surgeons in 2018 supporting a change in regulation to allow more use of it [32]. However, there has reportedly been a large surge in telemedicine across all forms of veterinary practice, borne out of necessity during the pandemic [33]. We have heard, again, anecdotally, that there has been greater use of photos or livestreaming of racehorses to remote veterinary surgeons for diagnosis, and less frequent physical visits by veterinarians to training yards. The Federation of Veterinarians of Europe (FVE), of which the UK is a member, defines this type of practice (veterinary telemedicine) as, *“the exchange and use of animals’ health information through technological platforms between a vet and a recipient (client, vet or other health professionals) in the context of a vet-client-patient relationship (VCPR)”* [34]. It is seen as one of the emerging areas of practice in the veterinary sector, but is not without its critics [35]. A VCPR is normally the physical interaction among vet, their clients and their patients, for the benefit of the patients. Telemedicine thus extends to the provision of veterinary services by video-link, text, instant messaging or telephone, or by any other remote means [36], although vets have been exchanging information with clients and colleagues, by means other than face-to-face interaction, for decades.

The relationship between racehorse trainers and their veterinary surgeons is integral to both protecting horse welfare and success on the racetrack. Racehorses typically benefit from intensive veterinary involvement in preventing health and welfare problems and at an early stage of injury or illness. Training yards have been shown to experience a mean of three horse-related accidents per year, 50% of which required veterinary treatment [37]. Research by Butler et al. [38] found that racing stakeholders perceived health problems to be the main challenge to racehorse welfare, but that veterinary care was also an area likely to generate new innovations benefiting equine welfare [30]. 

Thus, the aim of this qualitative study was to understand how the disruption caused by the COVID-19 pandemic may have brought about beneficial changes to racehorse veterinary care. In order to try and meet our aim our research, questions focused on gaining an understanding of the relationship between vets and racehorse trainers, to identify changes in the relationship and practices brought about by COVID-19 and to identify suitable methods for the dissemination of beneficial innovations, developed during the COVID-19 pandemic.

## 2. Materials and Methods

In order to fulfil the aim of the study, we used a qualitative methodology. This type of approach provided the opportunity for the collection and analysis of an in-depth and contextualised exploration of our participants thoughts, feelings and perceptions and how these might provide some answers to our research questions.

The methodology for the study was informed by an epistemology that was influenced by the main researcher’s views that the situation being researched would illustrate the interrelationship between individuals, groups and the organisations they were located within. The methods chosen, snowball sampling, semistructured interviews and a critical realist social constructionist approach to thematic analyses [39,40], would provide the space for participants to be active contributors, and for the researchers to imagine how the perceptions of the vet–trainer relationship might be constructed, through a shared assumption about reality, about “real life” [41]. It would be interesting therefore to consider, had travel restrictions and “stay at home” messages been lifted how the knowledge that would have been constructed might have varied from that constructed within this study.

### 2.1. Research Methods

#### 2.1.1. Participant Recruitment

One of the authors (L.U.) is employed by the British Horseracing Authority, the governing body for horseracing, and identified training yards that were diverse in size (but focussing on larger yards with more veterinary need). She was able to identify equine vets and trainers who were known to her in a professional capacity who were willing to participate in the research. 

The first author (D.B.) has worked and is still closely involved in the racing industry and was able to draw upon her biography and personal contacts to recruit trainers and vets as research participants. Using personal contacts in this manner raises the question of epistemological privilege [42]. For instance, her biography gives her a lived familiarity with some of the participants being studied, her tacit knowledge informing the research so producing a different knowledge that would be available to an outsider, a researcher who does not have an intimate knowledge of the group being researched, prior to the researcher’s entry into the group [43,44]. As a researcher in this position it has been important to be reflexive, to move across social and conceptual boundaries, to be both subject and object [45] and to practice “epistemological vigilance” [46]. In other words, to reflect on D.B.’s social contexts and the conditions in which they are produced, her ways of thinking and prejudices that may colour her view of the world. The author’s epistemic beliefs, that is, her perceptions regarding the nature of knowledge and knowing (of the racing industry per se), provided justification of the methods used in the study, producing data and analyses from which knowledge can be created.

This method of participant recruitment typically referred to as snowball sampling is a commonly employed nonprobability sampling method [47] used in qualitative research, typically in medical research and various social sciences such as political science, human geography and sociology [48]. The most frequently employed definition of snowball sampling is “of a sampling method in which one interviewee gives the researcher the name of at least one more potential interviewee, so the sample grows especially if more than one referral per interviewee is provided.” Snowball sampling does not rely on a sampling frame, that is, a list of the members of the population to be studied [49,50].

As a method, snowball sampling can work well when using face-to-face interviews as it can generate the trust that is claimed is required in order to gain referrals [51]. In this study, this was not possible, but D.B. was able to use her “insider knowledge” [43,52] to telephone and email participants to see if they would be willing to be interviewed and to suggest other participants from within their occupational field who we could hopefully interview. Snowball sampling is also useful as a sampling approach for groups who are otherwise hard to reach [53]. However, whilst this form of snowball sampling is very useful in gaining access to certain groups, researchers need to be aware that they inherit the experiences, dispositions and attitudes of each individual as to who are the next suitable interviewees [50].

Once participants were identified, they were either emailed or telephoned to arrange a short interview. Prior to the interview, all participants were sent, via email, a participant information sheet and a consent form to sign and return, which informed them of their right to withdraw from the study. At the end of the interview, we asked each participant if they could suggest other trainers or vets they know who may be willing to be interviewed. 

Table 1 and Table 2 show the diversity in size and type of training yards and veterinary practices, respectively.

The vets (see Table 2) who were recruited were from largely equine practices of differing sizes and location and dealt mainly with racehorses in training.

#### 2.1.2. Data Collection

Semistructured interviews consist of a dialogue between researcher and participant and require a relational focus, by interacting and actively listening to participants. The interviews should be guided by a flexible interview schedule that provides the space for follow-up questions, probes and comments [54].

Interview question topics comprised the trainer–vet relationship (pre COVID-19 pandemic); changes in trainer–vet relationship during the first “lockdown”; beneficial changes for racehorse veterinary care; problematic changes for racehorse veterinary care; management changes enforced during the first “lockdown”; innovative features of veterinary advice, diagnosis and treatment (vets) and information sources of new veterinary practices (racehorse trainers). 

In this study, qualitative semistructured interviews were conducted with ten racehorse trainers (four female and six male) and ten male veterinary surgeons from September 2020 to January 2021. Three of the interviews were carried out by videoconferencing, the other 17 by telephone. It was decided that, in order to give participants the opportunity to think about the areas we were wanting to investigate, we would email the interview schedule out a few days before the interview was due to take place. There were a number of reasons we decided to take this approach. It was in part to meet some of the responses we had when arranging video conferencing and telephone interviews when participants reported that they did not now have time, “*they would be in between calls”* (vet) and “*they would have nothing to say*…” (trainer). Letting them see the interview schedule before the interview we thought would give them the time to reflect and remember, in their own time, how COVID-19 and the first “lockdown” period (23 March–12 May 2020) had affected their work. One participant emailed a completed interview schedule when phone connectivity was poor and we were unable, for much of the interview, to hear what she was saying. Prompts were used when carrying out the interviews to keep the interviews focused on the topics being investigated and to try and keep the dialogue flowing, for example, when asking trainers to describe the relationship they had with their vet, we used prompts to find out how often the vet visited the yard, what type of issues would they advise on and if there were any “virtual interactions”. 

As a method of data collection, desk-bound data collection was the only viable option we could take at the time. It does have advantages in that video or telephone interviews overcome the barriers of geography, can provide an increased level of privacy and generally cost less with a reduced cost of failure should the interview need to be rescheduled, as some of the interviews for this study had to be.

### 2.2. Data Analysis

The three videoconference and 17 telephone calls were audio-recorded using either Teams or Rev call recorder, respectively, transcribed verbatim and analysed using a critical realist constructionist thematic analysis. Critical realists are ontological realists, whereby it is assumed that the data are able to tell us something about reality although are not viewed as directly mirroring it [55,56]. It is epistemologically relationist, that is, different methods provide different perspectives on reality [57]. It offers the opportunity to explore the discursive constructions of our participants as well as providing a further layer of detail in going beyond the text, setting what has been said in a broader historical, cultural and social setting [58].

Social constructionism primarily concerns itself with how knowledge is constructed and mainly takes the position that this occurs in social processes between us, taking a critical stance toward taken-for-granted knowledge [59,60]. The basic contention of the constructionist argument is that reality is socially constructed by and between the persons who experience it [61]. It is a consequence of the context in which the action occurs and is shaped by the cultural, historical, political and social norms that operate within that context and time. Reality can be different for each individual based on unique understandings of the world and our experience of it [62]. Reality in this case is completely subjective and need not be something that can be shared by anyone else, but at the same time it is independent of the person living it. 

Putting the two frameworks together offered the opportunity to incorporate participants’ discourses regarding vet–trainer relationships pre and post COVID-19, and to consider whether there were any beneficial innovations brought about during this period of unprecedented change and shifting regulatory control. Whilst the research questions aimed to produce knowledge about human experience, the objective was to understand what may have given rise to these experiences, that is, the contextual impact of the disruption COVID-19 had on participants’ occupational relationships and how the disruption created a shift in their relationships and practice.

Thematic analysis provided the most suitable methodological framework as theories can be applied to it flexibly [40,63,64] without single a priori theoretical assumptions about what may be learned from the data [65]. As such, the researcher is able to interpret individuals’ accounts of their experiences, their perspectives of working during the pandemic, as well as generating unanticipated insights [40]. As a qualitative method, it is an iterative process that gives the researcher the opportunity to ask whether a set of data answers the research questions in a meaningful way [66].

An inductive approach was undertaken to capture and identify patterns within the data, in order for themes to be driven by, and strongly linked to, the data [40,64], in line with the epistemological position. Themes, however, do not “emerge” from the data, and do not directly represent the spoken word [67]. Rather, themes were actively constructed by the researcher, informed by the literature (although there was little, if any, written on the topics being researched during this period) as well as the researchers’ beliefs, assumptions and experiences [68]. 

We have presented the steps we took to analyse the data as linear and distinct. However, it was a reflective and at times convoluted process that involved a constant re-evaluation of codes, themes and the raw data.

1. The data sets (transcripts) were first read and re-read independently by both authors to familiarise themselves with the data, colour highlighting the data sets to provide a rough outline of any similarities, patterns and ideas that may relate back to the research questions. The vet and trainer transcripts were analysed as independent cohorts to start with.
2. Rudimentary labels from each cohort were produced with handwritten links to textual extracts from within the data sets. Using the data sets, the authors compared the rudimentary labels each had generated. Initial codes were sketched out using the labels at the latent or semantic levels of analysis for each cohort [40].
3. Using the research questions as guides, themes (coherent and meaningful patterns that capture and summarise a meaningful pattern in the data) were identified. This was an active and at times messy process.
4. The draft themes were reviewed and checked against the codes and the full data set.
5. Four themes were decided upon. These were good working relationship pre COVID-19, little or no change in the vet–trainer relationship (first “lockdown”), beneficial changes to veterinary care and poor connectivity and its effect on technology. Table 3 outlines the themes, codes and their relationship to the research questions.

## 3. Results

The aim of the study was to capture any beneficial changes to racehorse veterinary care implemented during the (first) COVID-19 “lockdown” period. The study focused on gaining an understanding of the relationship between vets and racehorse trainers (research question, RQ 1,2) to identify changes in the relationship and practices brought about by COVID-19 (RQ 2) and to identify any beneficial innovations developed during the COVID-19 pandemic (RQ 3). Using a social constructionist framework has provided the opportunity to illustrate how participants have, through the data collected, tried to make sense of and interpret their own realities, their multiple subjectivities experienced during the first “lockdown” period. We identified four themes, as illustrated in Table 3.

## 4. Results and Discussion

### 4.1. Good Working Relationship Prior to COVID-19

In order to put the vet–trainer relationship in context, trainers either had routine visits every week or would use some form of communication channels, typically WhatsApp^™^, to discuss any problems they may have with their horses first before calling “their” vet out. Vets felt it was important to maintain links with the yard, especially in training centres where there was a perceived high degree of competition between veterinary practices. 

When participants discussed the trainer–vet relationship prior to lockdown, the subject that came most to the fore as a theme was the importance of having a good working relationship, both prior to and during “lockdown”:

*“I can ring our vet about a horse that has a problem we have not really seen before. We had one horse who had a horrible rash, like ringworm but it wasn’t as we know what that is. I rang [the vet] and he suggested what we might do, which we did. We don’t have a routine weekly visit and try not to call him out, cost I suppose, to the owner being one reason. He [vet] has said he is happy for us to ring him directly, on his mobile and if it’s an emergency we do and he tries to get to us as soon as he can.”*

*(racehorse trainer)*

Whilst for some trainers the trainer–vet relationship was a reciprocal relationship, for others it was something that had to be worked upon and was vital if their yard was to be successful and horses kept fit and healthy. As one trainer stressed,

*“it took a while to build up a good working relationship with the vet, a relationship that could be trusted.”*

*(racehorse trainer)*

Within the good working relationship theme was the notion of trainers being,

*“professional people, who know their horses and are used to dealing with most ailments, in the same way farmers are with their livestock. It’s different with leisure riders, they often don’t have the depth of experience, that knowledge. I know if I am called out to a yard to look at a horse then it must be something quite serious or an on-going lameness for example that isn’t improving.”*

*(vet)*

The perception of the trainer as a professional is one that on reflection is difficult to define. It could be someone who can be characterised as no more than a list of characteristics and behaviours, for example, as an individual who is competent, experienced, skilful and knowledgeable in their chosen occupational field. However, racehorse trainers by the nature of their occupation are typically paid to train horses that belong to other people and are licensed to do so by the BHA. They are thus part of an autonomous body and as such need to ensure they have both tacit and formal knowledge of equine welfare, health and management. We would suggest that when compared to a leisure rider, the motivation of a trainer is extrinsically driven in that their success is driven by wider external factors such as training winners, which involves making sure the horses they train are kept fit, healthy and sound, and as such the vet regards the occupation of trainer in this light. 

Comparing the trainer with a leisure rider is not dissimilar to comparing a professional sports player with an amateur, someone who engages with a sport on an unpaid rather than a professional basis. That said, whilst a leisure rider may ride for pleasure and not economic gain, it does not necessarily mean they are unknowledgeable, rather their motivation to ride is more intrinsic, that is, for pleasure and enjoyment, maybe as a hobby, not as a form of employment [69].

Whilst vets perceived trainers as “professionals”, there was the sense, a thread running through the data, that the vet–trainer relationship was a fluid and at times uncertain one. Vets were aware, in racehorse training areas such as Newmarket or Lambourn, that the vet–trainer relationship can be a tenuous one with an element of competition between practices. Trainers will switch vets:

*“if you can’t meet a trainer’s needs, their horses are not running well in spite of what you may have suggested, or so and so practice “cured” a fellow trainer’s best horse. There is always the other practice who seem more digitally savvy, seem to have better technology, seem to have better outcomes in terms of [horses] performance and are always cheaper.”*

*(vet)*

A view such as this runs counter to what some trainers expressed about building a good working relationship with their vet, and as the following quote illustrates, a dichotomous relationship can exist with regard to the vet–trainer relationship. It is perceived as one that takes time and trust to develop, in having confidence in the vet’s ability and knowledge, and possibly parallels the opinions held by farmers when a vet is perceived as not knowing how to interact with livestock or does not appear to understand the minutia of livestock farming practice [70].

*“It takes me a long time to get confidence in a vet. I think they are generally far too keen to use technical equipment, frequently too keen to rely on that equipment when good old fashioned ‘stockmanship’ is what I require when assessing a horse’s lameness, diagnosis and prognosis. Scans, X-rays and scopes sometimes confirm or clarify what you think you know, but they certainly do not cure them, and frequently, if a vet who has just met the horse and does not appreciate the full history of the horse, comes in and relies too much on technical machines, they end up not even treating the right ailment… Several vets have let me down, even older more mature vets. I can name on one hand the vets I really rate, and only two live near me, therefore I would have to be desperate to contact a vet practice I did not personally know.”*

*(trainer)*

### 4.2. Little/No Change in Vet/Trainer Relationship (during First Lockdown)

The second theme identified is that of little or no change in the trainer–vet relationship during the first “lockdown”. As a theme, it helps demonstrate how using a critical realist social constructionist approach to thematic analysis helps to uncover the subjective perceptions of the trainers to the working relationship they had with their vet during this period. For example, when analysing the transcripts, one of the most common expressions used by trainers was that of little or no change. Participants routinely reported that they had experienced little or no change to the trainer–vet relationship and practices during the first “lockdown” period.

*“We carried on as normal …no change really so I’m not sure what else I can tell you…” (trainer) and “we hardly needed a vet and don’t get him out very much anyway, only when we need. I don’t think, can’t remember doing any different to what we did before with any veterinary care when “lockdown” stopped everything.”*

*(trainer)*

As there was no racing during the first “lockdown”, many trainers turned their horses out, *“there were only three horses in, we turned all the others out who all behaved and didn’t hurt themselves. With no racing there wasn’t as much need for [veterinary] weekly visits anyway” (racehorse trainer)*.

During the first “lockdown” period, vets were limited to only carrying out visits for emergencies [29,71], and as the previous quote alludes, with fewer horses in training and no racing, veterinary care was not needed as much. Trainers’ perceptions were such that “*there was no change*”. Nevertheless, for the horses in training and for some out of training, there appears to have been an increase in the use of remote veterinary consults by trainers, with trainers setting up dedicated WhatsApp™ groups (a multi-platform messaging app) between themselves, senior members of staff and their vet during the “lockdown” period, so they had a remote connection to their vet, which had been agreed between vet and trainer.

On reflection at a macro level, trainers implicit perceptions of the trainer–vet relationship was business as usual, yet at a micro level, running alongside the theme of little or no change, was the third theme, when COVID-19 restrictions were in force, more remote consultations took place using images or videos as well as telephone consults, viewed favourably by both trainers and vets, demonstrating how subtle yet explicit changes occurred within the trainer–vet relationship during the “first” lockdown. 

### 4.3. Beneficial Changes to Veterinary Care during the First “Lockdown”

During this perceived period of little or no change in the trainer–vet relationship, there was a greater emphasis put on the use of telemedicine by both cohorts, when face-to-face interactions at the yard were limited. 

Using telemedicine, as previously defined, was viewed as a useful innovation that could be used in aiding and benefitting veterinary care:

*“Lockdown meant we could not have our weekly visits (from the vet). We still had some of our flat horses in work and overall, ringing him [vet] or sending a picture seemed to work reasonably well. If we had a horse who was not coming sound we would film the horse trotting up and send that through. If then he [vet] thought the horse needed seeing he would come out.”*

*(racehorse trainer)*

In this study, telemedicine gave the opportunity to assess a case, especially at the start of lockdown when visits were limited to emergencies only. Vets reported they experienced an increase in the amount of remote consultations they were doing. Their experiences were substantiated by the results of a COVID-19 Impact Study carried out by the British Equine Veterinary Association (BEVA). The report records how 75% of BEVA members were undertaking more remote consultation [72]. Almost all respondents (98.39%) had used remote consults to give advice as well as triage a case, that is, deciding the order, seriousness and treatment of a case, highlighted by vets in this study:

*“I would ask the trainer or senior staff to video the horse in question. If it was a decent recording it was useful to look at before going out to see the horse. It gave me time to maybe run through treatment options in my head. For example, if it was a video of a lame horse say, it wouldn’t be something I could give a definite diagnosis to from the video, but at least I got an impression of how that horse was moving on that day when the trainer got in touch.”*

*(vet)*

In this manner, images and videos were said to have been useful adjuncts when triaging a case, one of the advantages outlined in RCVS research on telemedicine [36]. Other advantages include improved access to veterinary services, making a diagnosis more efficient and convenient, additional welfare benefits as more animals are seen by vets, together with being a chargeable service [36,72]. Vets’ perceptions were that trainers preferred a more hands on approach when examining a horse, injuries, for instance, can be looked at and dealt with more quickly. There is also the fact that some practices have been charging for remote consultations [72], which on occasion trainers found harder to justify, especially when some of the COVID-19 restrictions were temporarily lifted:

*“I will be so glad when things can return to normal. We all want to do the best for our horses, that goes without saying, but when my secretary showed me the invoice from our vet and there was a charge for having a chat on the phone about a lame horse then getting one of my staff to send images which we then dealt with, once he [vet] had looked at them, well, I wasn’t very impressed. All the money they [vet practice] get from us too.”*

*(trainer)*

In May 2020, following guidance from the British Equine Veterinary Association, many equine veterinary services could be held outside. Non-emergency veterinary activities, with appropriate facilities, could be performed as long as COVID safety precautions were followed [71]. The return to non-emergency consults was viewed as being able to,

*“… actually look, observe and assess a case. Yes, ok, at the moment it is harder, but when things are managed, social distancing, wearing masks and having only one other person there if needed I feel it is far better in terms of welfare. I think trainers prefer the hands-on approach anyway.”*

*(vet)*

The quotes included earlier provide a snapshot of how, during the first “lockdown”, there were beneficial changes that were introduced to veterinary care, which vets and trainers saw as important measures in maintaining equine welfare. It would be interesting to see, however, if these changes are permanent, if trainers have continued to use some of the communication channels outlined in parallel with a face-to-face consultation as an extra diagnostic tool. Vets, as we have described, felt they needed to look at a horse, to touch, feel and observe a horse’s demeanour and behaviour, but also found the use of telemedicine useful. 

Participants, both trainers and vets, referred to the Weatherby’s E-Passport Vaccination app (vaccination app) (Weatherbys, Wellingborough, UK.) introduced in May 2020 as a management change that was introduced during the first “lockdown” [73,74,75]. The vaccination element of the app was launched earlier than expected as a compliance measure, to help combat the spread of COVID-19 at a race meeting when racing resumed. Participants also described it as an innovative tool for the future that could be beneficial in terms of veterinary care once the other elements of the E-Passport are operational, said to be in 18 months’ time [74,75]. Once completed, the app will have other functions such as recording information on identification, medications given, equine movement and ownership, which has the potential to improve traceability and monitor the health and welfare of horses in training.

Trainers are now required to scan in and upload a horse’s historical vaccination records from the paper-based passport, which are then checked and approved by the BHA. Future vaccinations have to be uploaded directly into the vaccination app. The app reduced the need for raceday staff and stable staff to be in close proximity of each other at the racecourse stable entrance and removed the risk of handling a potentially contaminated object such as a horse passport, which historically was checked manually at the racecourse [74]. In terms of a management change, it was viewed as being beneficial as trainers no longer have to worry about passports being mislaid, lost when taken racing or vaccinations being out of date, thus making the trainer liable for a fine. As one trainer explained,

*“the vaccination app has helped us as it has meant we don’t have to worry now about passports being forgotten, or worse lost when they get taken with the runners to a race meeting in case they have to be checked. You don’t know ‘til you get there if they [raceday staff] might want to look at a passport and if they do need to check, do a trot up for instance, the vets come after you have the horse in their stable and have got them hopefully settled.”*

*(trainer)*

With regard to a beneficial change to veterinary care, the benefits at present appear to be greater for the personnel involved than the horses. Participants discussions around the app during interviews provide a further layer of discursive detail that goes beyond the text that participants have constructed and as such is a consequence of the context on which the perception was based. 

### 4.4. Poor Connectivity and Use of Technology

Poor connectivity was a thread that ran through much of the data both for trainers and for vets. In areas where connectivity was poor, uploading vaccination data into the Weatherby’s E-Passport Vaccination app turned into a very long-winded and time-consuming operation, almost impossible at times:

*“I found myself having to sit in the car at the end of the road about half a mile from the [racing] yard with a bag full of passports as that was the only place I could nearly guarantee I would get enough signal to upload the vaccination records. It took ages. It has been said that this region will be one where 5G will be trialled, so I hope they hurry up and get a move on as at the moment our connectivity to anything is pretty poor.”*

*(trainer)*

Poor connectivity made uploading and downloading videos and images difficult and slow, which sometimes hindered being able to triage a case, especially when vets were constrained to emergencies only [29,71]. When images or videos were distorted or, as was mentioned, where connectivity was extremely limited, remote consultation proved almost impossible, one of the disadvantages highlighted in the use of telemedicine [36].

*“On a couple of occasions, when trainers had sent images through and videos they wanted me to look at in case the horse needed looking at, as an emergency, I struggled to get the videos to download properly. I found myself having to drive to places where I knew I would get good signal or drive to the practice as the internet was faster, all adding time to being able to assess a case.”*

*(vet)*

Connectivity was not the only issue when videos and images were used as assessment, of a cut, for example, and it was suggested that for remote consultations to be more useful, training should be given, either at seminars or as webinars, on taking images that can be interpreted by the vet:

*“Sometimes I would be sent an image of a horse with a cut and the trainer would want to know if it needed stitching which would have meant going out to the yard during ‘lockdown’, as an emergency. The images weren’t always the best, they would either be too far away or make the wound look bigger, wider than it was or even from a funny angle. They would be blurred and difficult to know where or what part of the horse had been cut. I would end up having to phone and guide them through taking the images then sending them through. Great when the signal was good, not if the signal was poor.”*

*(vet)*

If telemedicine is to be used as a complementary assessment tool, in order to take advantage of its use, we would recommend that rural connectivity is greatly improved. Participants within this study have drawn attention to how poor access to core digital infrastructure can be disruptive. The same has been shown within wider society, for instance, in higher education [75,76] and as a potential downside to a country’s gross domestic product (GDP) [77].

### 4.5. Limitations to the Research

It goes without saying that the ongoing pandemic changed the nature of the research, but its effects were not insurmountable. In September, as COVID-19 cases started to rise again, restrictions were tightened regarding the mixing of households. With COVID-19 tier regulations brought in during October, it was decided that interviews should be carried out, either by telephone or by video conferencing where possible, a scenario that had been written into the research proposal although it had been thought originally that by October 2020 COVID-19 restrictions would have allowed greater face-to-face interaction. 

It would have been useful if we could have widened the scope of the study by interviewing a larger cohort of vets and trainers, although the ongoing “lockdown” restrictions would have kept any interviews literally in house.

One of the themes identified in the study was poor connectivity in certain areas of the UK. A similar theme could be drawn out of this study with both authors working remotely from home, using videoconferencing to communicate, which, at times was limited by poor connectivity.

## 5. Conclusions

In partnership with the BHA, the aim of this study was to identify any beneficial changes to racehorse veterinary care that occurred due to the implementation of the first COVID-19 “lockdown” restrictions. In March 2020, vets in the UK were restricted to only treating emergencies at the beginning of the first “lockdown”. In May 2020, non-emergencies could be treated, but with racing suspended from 18 March 2020 to 1 June 2020 for flat racing and National Hunt racing starting in July 2020, there were fewer horses in training that needed veterinary treatment. Vets reported that there was very little change in their working relationship with trainers, although there was an increase in the use of other forms of technology as well as the telephone, which included images and videos, (telemedicine) useful adjuncts in triaging a case, complementary to but not viewed as a replacement for face-to-face examinations in an emergency. The use of telemedicine was hampered in some cases by the lack of connectivity as well as poor images or videos.

The use of technological innovations, in this instance, the vaccination app, was introduced earlier than planned. Launched in May 2020 during “lockdown”, it reduced the need for human contact at race meetings. The vaccination app was perceived quite widely, by trainers and vets alike, as a useful potential management tool in combating the spread of COVID-19, although it would be difficult to see it as a beneficial change to veterinary care until the E-Passport is launched. In order to take advantage of more advanced technology, we would recommend that rural connectivity is greatly improved across the UK.

## Figures and Tables

**Table 1:** Diversity in size and type of training yards.

Trainer	Type of Yard Combined/Flat/NH Licence	Number of Horses in Training pre COVID-19	Number of Horses in Training during Lockdown
Trainer 1	Combined licence	35	4
Trainer 2	Combined licence	40	2
Trainer 3	NH licence	9	2
Trainer 4	NH licence	92	8
Trainer 5	Flat licence	80	80
Trainer 6	Dual licence	86	40
Trainer 7	NH licence	40	30
Trainer 8	Combined licence	104	60
Trainer 9	Flat licence	30	20
Trainer 10	Combined licence	40	20

**Table 2:** Practice location, number of vets employed and type of practice.

Vet	Practice Location	No. of Equine Vets Dealing with Horses in Training and Type of Practice
Vet 1	Lambourn	8 equine vets, equine hospital
Vet 2	Lambourn	7 equine vets, equine hospital plus companion animal surgeries
Vet 3	Newmarket	12 equine vets, equine hospital
Vet 4	Gloucestershire	11 equine vets, equine hospital
Vet 5	Worcestershire	3 equine vets, equine hospital
Vet 6	Gloucestershire	11 equine vets, equine hospital
Vet 7	Yorkshire	4 equine vets, farm and small animal practice
Vet 8	Yorkshire	4 equine vets, farm and small animal practice
Vet 9	Newmarket	15 equine vets, equine hospital
Vet 10	Shropshire	3 equine vets

**Table 3:** Themes, codes and their link to research questions.

Themes	Codes	Research Questions
Good working relationship pre COVID-19	No real virtual interactions Use of WhatsApp^™^/Phone calls Trust in vet Trainer as a “professional” person able to deal with non-emergencies Trainers seem to prefer face-to-face consults	RQ 1,2
Little/No change in vet–trainer relationship (during 1st lockdown)	No difference in the vet–trainer relationship Less face-to-face interactions with vet Increase in WhatsApp^™^/Phone for vet consults Fewer visits made	RQ 2
-	Visits for emergencies only	-
-	Fewer equine injuries	-
Beneficial changes to veterinary care	Telephone/Images/Videos were used for remote triaging	RQ 2,3
Poor connectivity and use of technology	Phone signal very poor in some areas	-
-	Images unclear	-
-	Training for vets and trainers on producing usable images of, for example, a wound, or video of lameness	-
-	Vaccn’app dependent on good connectivity	-
-	Vaccn’ app initially very time consuming, but should be worth it to record medications given	-

## Data Availability

The data presented in this study are available on request from the corresponding author. The data are not publicly available to protect study participant privacy.
